# Activation of GPER1 by G1 prevents PTSD‐like behaviors in mice: Illustrating the mechanisms from BDNF/TrkB to mitochondria and synaptic connection

**DOI:** 10.1111/cns.14855

**Published:** 2024-07-11

**Authors:** Lixia Chen, Yang Zhang, Zisheng Wang, Zhengrong Zhang, Jingji Wang, Guoqi Zhu, Shaojie Yang

**Affiliations:** ^1^ Center for Xin'an Medicine and Modernization of Traditional Chinese Medicine of IHM, Key Laboratory of Molecular Biology (Brain diseases) Anhui University of Chinese Medicine Hefei China; ^2^ Acupuncture and Moxibustion Clinical Medical Research Center of Anhui Province The Second Affiliation Hospital of Anhui University of Chinese Medicine Hefei China

**Keywords:** brain‐derived neurotrophic factor, G protein‐coupled estrogen receptor 1, mitochondrial function, post‐traumatic stress disorder, synaptic function

## Abstract

**Background:**

G1 is a specific agonist of G protein‐coupled estrogen receptor 1 (GPER1), which binds and activates GPER1 to exert various neurological functions. However, the preventive effect of G1 on post‐traumatic stress disorder (PTSD) and its mechanisms are unclear.

**Objective:**

To evaluate the protective effect of G1 against synaptic and mitochondrial impairments and to investigate the mechanism of G1 to improve PTSD from brain‐derived neurotrophic factor (BDNF)/tyrosine kinase receptor B (TrkB) signaling.

**Methods:**

This study initially detected GPER1 expression in the hippocampus of single prolonged stress (SPS) mice, utilizing both Western blot and immunofluorescence staining. Subsequently, the effects of G1 on PTSD‐like behaviors, synaptic, and mitochondrial functions in SPS mice were investigated. Additionally, the involvement of BDNF/TrkB signaling involved in the protection was further confirmed using GPER1 antagonist and TrkB inhibitor, respectively.

**Results:**

The expression of GPER1 was reduced in the hippocampus of SPS mice, and G1 treatment given for 14 consecutive days significantly improved PTSD‐like behaviors in SPS mice compared with model group. Electrophysiological local field potential (LFP) results showed that G1 administration for 14 consecutive days could reverse the abnormal changes in the gamma oscillation in the CA1 region of SPS mice. Meanwhile, G1 administration for 14 consecutive days could significantly improve the abnormal expression of synaptic proteins, increase the expression of mitochondria‐related proteins, increase the number of synapses in the hippocampus, and ameliorate the damage of hippocampal mitochondrial structure in SPS mice. In addition, G15 (GPER1 inhibitor) and ANA‐12 (TrkB inhibitor) blocked the ameliorative effects of G1 on PTSD‐like behaviors and aberrant expression of hippocampal synaptic and mitochondrial proteins in SPS mice and inhibited the reparative effects of G1 on structural damage to hippocampal mitochondria, respectively.

**Conclusion:**

G1 improved PTSD‐like behaviors in SPS mice, possibly by increasing hippocampal GPER1 expression and promoting BDNF/TrkB signaling to repair synaptic and mitochondrial functional impairments. This study would provide critical mechanism for the prevention and treatment of PTSD.

## Graphical abstract

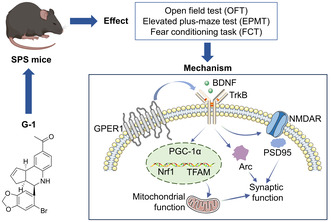

## 1 INTRODUCTION

Post‐traumatic stress disorder (PTSD) is a heterogeneous mental disease that occurs and persists after an individual experiences a serious threatening or catastrophic event.^1^, ^2^ The primary symptoms include traumatic memory flashbacks, sustained hyperarousal, and avoidance of trauma‐related factors.^3^ Statistically, approximately 70% of the global population has encountered trauma, with around 6% of those developing PTSD.^4^, ^5^ Research revealed a significant gender disparity in PTSD prevalence, with women being twice as susceptible as men to the disorder after similar traumatic events.^6^, ^7^ Evidence indicated that estrogen modulates fear and stress responses in females, including rodent models, making them more prone to PTSD or symptom aggravation during low estrogen periods.^8^, ^9^ In addition, it has been reported that flashbacks of traumatic memories are significantly more prevalent during the luteal phase (a period of low estrogen levels) in women who have been recently traumatized.^10^ However, the specific neurobiological mechanisms of PTSD remain unclear, with limited exploration into estrogen‐related therapies and strategies.

Fear memory abnormalities are a core feature of PTSD, and the hippocampus is thought to be involved in the formation, consolidation, and extinction of fear memories.^11^, ^12^ PTSD patients and various rodent models exhibit hippocampal structural atrophy, characterized by a decrease in volume.^13^, ^14^, ^15^ Meanwhile, it was found that fear memory abnormalities are associated with impaired synaptic function in the hippocampus.^16^, ^17^ Brain‐derived neurotrophic factor (BDNF)contributes to neuronal growth and synaptic function via tyrosine kinase receptor B (TrkB) receptor activation and is linked to psychiatric disorder pathogenesis.^18^, ^19^ Our prior researches indicated that BDNF/TrkB signaling inhibition was a crucial mechanism for the fear memory abnormalities in mice with PTSD‐like behavior.^20^, ^21^, ^22^, ^23^, ^24^ Mitochondria have a very positive role in regulating synaptic function, and some studies have reported mitochondrial dysfunction in patients with PTSD.^25^, ^26^ Peroxisome proliferator‐activated receptor γ coactivator 1‐α (PGC1‐α) is a crucial mediator of mitochondrial energy metabolism as well as a modulator of BDNF. We have previously investigated the mechanism of PGC1‐α in fear memory abnormalities in mice model of PTSD through modulation of mitochondrial function.^27^

The G protein‐coupled estrogen receptor 1 (GPER1), a seven‐transmembrane domain protein, mediates rapid estrogen responses and transcriptional regulation, contrasting with classical nuclear estrogen receptors ERα and ERβ.^28^ G1, a selective GPER1 agonist with a molecular structure akin to 17β‐estradiol, binds and activates GPER1, mediating various signals to safeguard neurons against oxidative stress, neuroinflammation, and apoptosis^29^, ^30^ with BDNF/TrkB as the downstream signal. Our previous study found that G1 could ameliorate the abnormality of fear memory in aging mice through the activation of GPER1 and the mechanism may be related to the activation of the BDNF/TrkB signaling.^31^ In this study, we explored the effect of G1on PTSD‐like behaviors in single prolonged stress (SPS) mice and investigated the mechanisms from the perspectives of hippocampal synaptic and mitochondrial functions, providing a new strategy for the prevention and treatment of PTSD.

## 2 MATERIALS AND METHODS

### 2.1 Preparation of SPS model

C57BL/6 male and female mice (2 months, 20–25 g)were purchased from Hangzhou Ziyuan Experimental Animal Technology Co., Ltd [production license No. SCXK (Zhe) 2019–0004]. The animals were housed at 20–24°C with 45%–65% relative humidity, subjected to 12 h light/dark cycles, with free access to water and food. The experimental protocol was supervised by the Ethics Committee of Anhui University of Chinese Medicine (Approval No. AHUCM‐mouse‐20210205). SPS was employed to induce PTSD‐like behaviors in mice, following previously established protocols.^20^ Briefly, mice were restrained for 2 h, then subjected to 20 min of forced swimming in a circular tank (30 × 50 cm, 22–24°C water temperature). Subsequently, they were anesthetized with 5% isoflurane, repeated upon awakening three times. Finally, mice received a single foot shock (2 mA, 2 s). Each step was separated by a 15‐min interval.

### 2.2 Animal experimental procedures and drug administration

#### 2.2.1 Experiment 1

Male mice were randomly divided into four groups (*n* = 6): control, SPS (D1), SPS (D7) and SPS (D14). The animals were anesthetized on days 1, 7, and 14 after SPS modeling, and then decapitated to collect hippocampus and whole‐brain samples.

#### 2.2.2 Experiment 2

Female mice were randomly divided into two groups (*n* = 3): control+OVX and SPS + OVX. Mice in each group were subjected to ovariectomy (OVX), and SPS modeling was performed 1 week later. 14 days after SPS modeling, animals were anesthetized and then decapitated to collect the hippocampus.

#### 2.2.3 Experiment 3

Male mice were randomly divided into five groups (*n* = 9): control, SPS, SPS + Pre‐G1 (administered 30 min before modeling), SPS + Acute‐G1 (administered immediately after modeling), and SPS + Continuous‐G1 (administered continuously for 14 days after modeling). G1 (HY‐107216, MedChemExpress) was administered via intraperitoneal injection at a dose of 5 μg/kg.^31^ Behavioral tests were conducted 14 days after SPS modeling. Thereafter, animals were anesthetized and then decapitated to collect the hippocampus.

#### 2.2.4 Experiment 4

Male mice were randomly divided into three groups (*n* = 4): control, SPS and SPS + G1. The administration and dosage of G1 were the same as in Experiment 3. After modeling, electrodes were implanted in the hippocampal CA1 region of mice in each group and were administered for 14 consecutive days. Brain waves in the CA1 area of the hippocampus were recorded on the 14th day.

#### 2.2.5 Experiment 5

Male mice were randomly divided into five groups (*n* = 9): control, SPS, SPS + G1, SPS + G1 + G15 and SPS + G1 + ANA‐12. G15 (HY‐107216, MedChemExpress, 185 μg/kg),^32^ and ANA‐12 (S7745, Selleck, 0.5 mg/kg)^20^ were administered by intraperitoneal injection. Behavioral tests were conducted 14 days after SPS modeling. Thereafter, animals were anesthetized and then decapitated to collect the hippocampus and whole‐brain samples.

### 2.3 Open field test (OFT)

The OFT was used to evaluate anxiety‐like behavior in mice. The experimental apparatus was an open open‐field box (40 × 40 × 40 cm), and the bottom was divided into 16 squares of 4 × 4 with the middle four squares considered to be the central area. The mice were placed in the center of the bottom of the box and allowed to explore freely in a quiet environment for 5 min, and the SuperMaze system (Shanghai Xinruan Information Technology) automatically recorded and analyzed their movements.

### 2.4 Elevated plus‐maze test (EPMT)

The EPMT was used to evaluate anxiety‐like behavior in mice. The elevated plus‐maze has two open arms (25 × 5 cm), two closed arms (25 × 5 × 5 cm), and a center area (5 × 5 cm). At the beginning of the test, the mice were placed in the center area facing the closed arm and allowed to move freely for 5 min. The SuperMaze system (Shanghai Xinruan Information Technology) automatically recorded their movement trajectories and analyzed the time spent in the open arm.

### 2.5 Fear conditioning task (FCT)

The FCT was used to evaluate fear memory in mice. There were four phases in FCT, which were adaptation period, training period, re‐exposure period, and extinction period. Experimental protocols for the four periods have been shown in our previous research.^27^ Meanwhile, in order to observe the fear memory extinction in mice, we defined an extinction coefficient^23^:

$$\text{Extinction coefficient}=\left(1-\left(\text{extinction period}\;\left(\text{freezing time}\right)/\text{first}\;3-\min\;\text{block in}\;\mathrm{re}-\text{exposure period}\;\left(\text{freezing time}\right)\right)\right)\times 100\%$$

### 2.6 Mouse ovariectomy

Female mice were anesthetized, and then, their abdomens were fixed and incisions were taken on both sides at the lower 1/3 of the midline of the back, followed by hair removal for skin preparation. The skin and dorsal muscle were incised to expose the cabbage‐patterned ovary. Thereafter, we ligatured the ovary below the fallopian tube using silk, excised the ovary, and then sutured the site. Bilateral oophorectomy was performed. The mice recovered for 1 week after surgery and then underwent SPS modeling.

### 2.7 Electrode implantation and local field potential (LFP) recording

The mice were anesthetized and fixed, and the skin was prepared on the top of the head, and the scalp was cut. The CA1 area of the hippocampus was labeled (AP: −2.3 mm, ML: 1.75 mm, DV: −1.55 mm), and the remaining three electrodes were fixed with silver wires. The electrode was slowly implanted into the CA1 area and fixed using dental cement. One week after recovery, LFP was recorded using the Medusa EEG EMG recording system (Bio‐Signal Technologies, Jiangsu), and the signals were categorized into Delta (0.5–4 Hz), Theta (4–8 Hz), Alpha (8–13 Hz), beta (13–30 Hz), and gamma (30–80 Hz) bands. The percent power spectrum of different frequency bands and power spectral density (PSD) were statistically analyzed using NeuroExplorer software, and the energy spectra were compared among groups.

### 2.8 Transmission electron microscopy

At the end of the behavioral tests, small pieces of tissue were isolated and excised from the CA1 region of the mouse hippocampus and fixed in 2.5% glutaraldehyde (pH = 7.4). The tissues were dehydrated and permeabilized, sectioned, and later stained with uranyl acetate and lead citrate. Changes in synapse number, mitochondrial morphology, and structure were observed using transmission electron microscopy.

### 2.9 Western blot

Mouse hippocampal tissues were homogenized with 100 μL RIPA buffer (containing 1% PMSF), then centrifuged for 5 min at 4°C and 12,000 rpm/min. At the end of centrifugation, the supernatant was aspirated, and the loading buffer was added and heated at 100°C for 10 min. After electrophoresis, the membranes were transferred with nitrocellulose (NC) membranes at 400 mA for 30 min. The NC membranes were removed and blocked with 5% skimmed milk powder at room temperature for 2 h. Membranes were washed with PBST (3 × 10 min). The primary antibody was incubated overnight at 4°C using the corresponding antibodies: GPER1 (1:1000, Bioss, bs‐1380R), BDNF (1:1000, ZEN BIO, 381133), p‐TrkB (1:1000, ZEN BIO, 381154), TrkB (1:1000, ZEN BIO, 385991), postsynaptic density protein 95 (PSD95, 1:1000, CST, 36233S), activity regulated cytoskeleton‐associated protein (Arc,1:1000, ZEN BIO, 120288), NMDA receptor subunit N2A (GluN2A, 1:1000, ZEN BIO, R381967), GluN2B (1:1000, Proteintech, 21,920‐1‐AP), mitochondrial transcription factor A (TFAM, 1:1000, Santa Cruz, I1022), nuclear respiratory factor‐1 (Nrf1, 1:1000, ZEN BIO, R25184), PGC1‐α (1:1000, ZEN BIO, 381615), GAPDH (1:1000, ZEN BIO, 380626). The membranes were washed with PBST and then incubated with peroxidase‐labeled goat anti‐rabbit/mouse IgG antibody (1:10,000, ZS‐BIO) at room temperature for 2 h. After washing, protein bands were visualized with enhanced chemiluminescence (ECL) solution and analyzed using ImageJ software.

### 2.10 Immunofluorescence staining

Tissues were placed in 4% paraformaldehyde and fixed at 4°C for 24 h and then dehydrated for 48 h using a 30% sucrose. Sections were performed using a freezing microtome with a slice thickness of 20 μm. The brain slices were washed with PBS (3 × 5 min), then placed in 0.3% Triton X‐100 in PBS for 10 min. The brain slices were washed, followed by blocking of the slices with 5% BSA containing 0.3% TrintonX‐100 for 30 min at room temperature and then incubated with GPER1 (1:100) and TFAM (1:100) at 4°C overnight. The brain slices were washed with PBS, after which they were incubated with FITC‐labeled anti‐rabbit IgG (1:100, ZS‐BIO, ZF‐0311) and Alexa Fluor 594‐labeled anti‐mouse IgG (1:100, ZS‐BIO, ZF‐0513) at room temperature for 2 h. DAPI was added 20 min before the end of incubation. The CA1 area of the hippocampus was observed and photographed with an FV3000 Olympus laser confocal scanning microscope. The average fluorescence intensity was analyzed using ImageJ software.

### 2.11 Statistical analyses

The experimental data were analyzed using GraphPad Prism 9.0 software, and statistical plots were expressed as means ± SEM. All data were first subjected to normality tests by Shapiro–Wilk test. For normally distributed data, independent sample *t*‐test were used for two groups, and one‐way ANOVA, two‐way ANOVA, or repeated measures ANOVA followed by Tukey's or Bonferroni's post‐hoc test for three or more groups. Non‐normal distributions were analyzed using non‐parametric test. Differences were considered statistically significant when *p* < 0.05.

## 3 RESULTS

### 3.1 Reduced hippocampal GPER1 expression is observable in SPS mice

**FIGURE 1:**
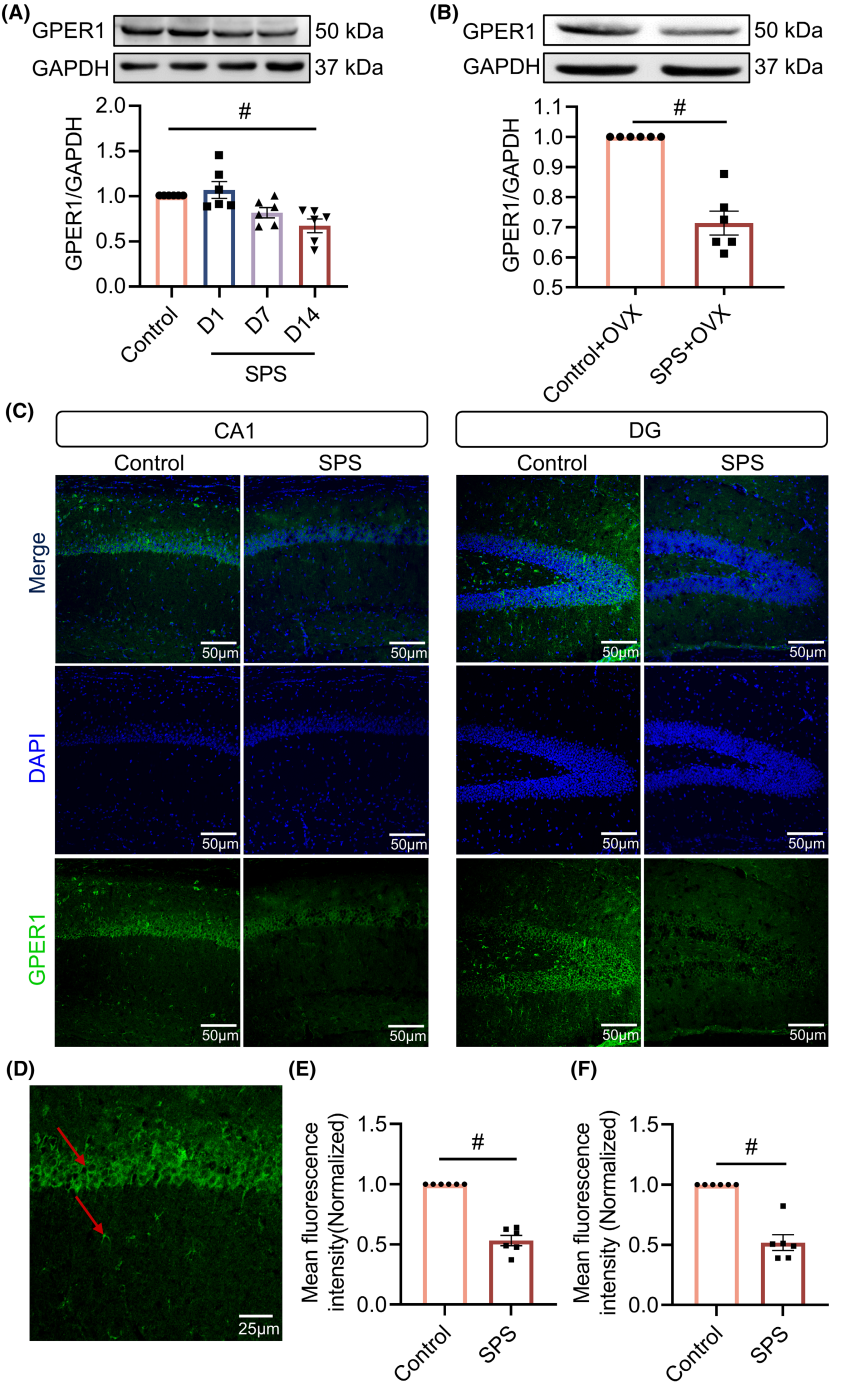
G protein‐coupled estrogen receptor 1 (GPER1) expression was reduced in the hippocampus of single prolonged stress (SPS) mice. (A) Representative blots and quantitative data for GPER1 in male mice (*n* = 3); (B) representative blots and quantitative data for GPER1 in female mice (*n* = 3); (C) typical immunofluorescence images showing GPER1 expression in the CA1 and DG region (*n* = 3); (D) enlargementGPER1 in CA1 region; (E) mean fluorescence intensity of GPER1 in the CA1 region; (F) mean fluorescence intensity of GPER1 in the DG region. Data are expressed as mean ± SEM. ^#^ *p* < 0.05 versus the control group (Tukey's test or *t*‐test).

We used Western blot to detectGPER1expression at days 1, 7, and 14 after SPS modeling, and the results showed that the levels of GPER1 were significantly reduced at 14 days after SPS modeling (vs. control, *p* < 0.05, *F*[3, 20] = 7.304; Figure 1A). At the same time, we used female OVX mice for SPS modeling and found that the GPER1 expression level was also significantly reduced at 14 days after modeling (vs. control+OVX, *p* < 0.05, *t* = 7.213, df = 10; Figure 1B). Thereafter, we chose male mice for subsequent experiments. Immunofluorescence staining was used to examine the distribution and expression of GPER1 in the hippocampus of control and 14 days after SPS modeling mice. The results showed that GPER1 was mainly distributed on neurons and glial cells (Figure 1C,D). The mean fluorescence intensity of GPER1 in the hippocampal CA1 region (vs. control, *p* < 0.05, *t* = 10.97, df = 10; Figure 1E) and DG region (vs. control, *p* < 0.05, *t* = 7.442, df = 10; Figure 1F) of the mice in the SPS mice was significantly reduced, consistent with the results of Western blot.

### 3.2 Continuous intraperitoneal injection of G1 improves PTSD‐like behaviors in SPS mice

**FIGURE 2:**
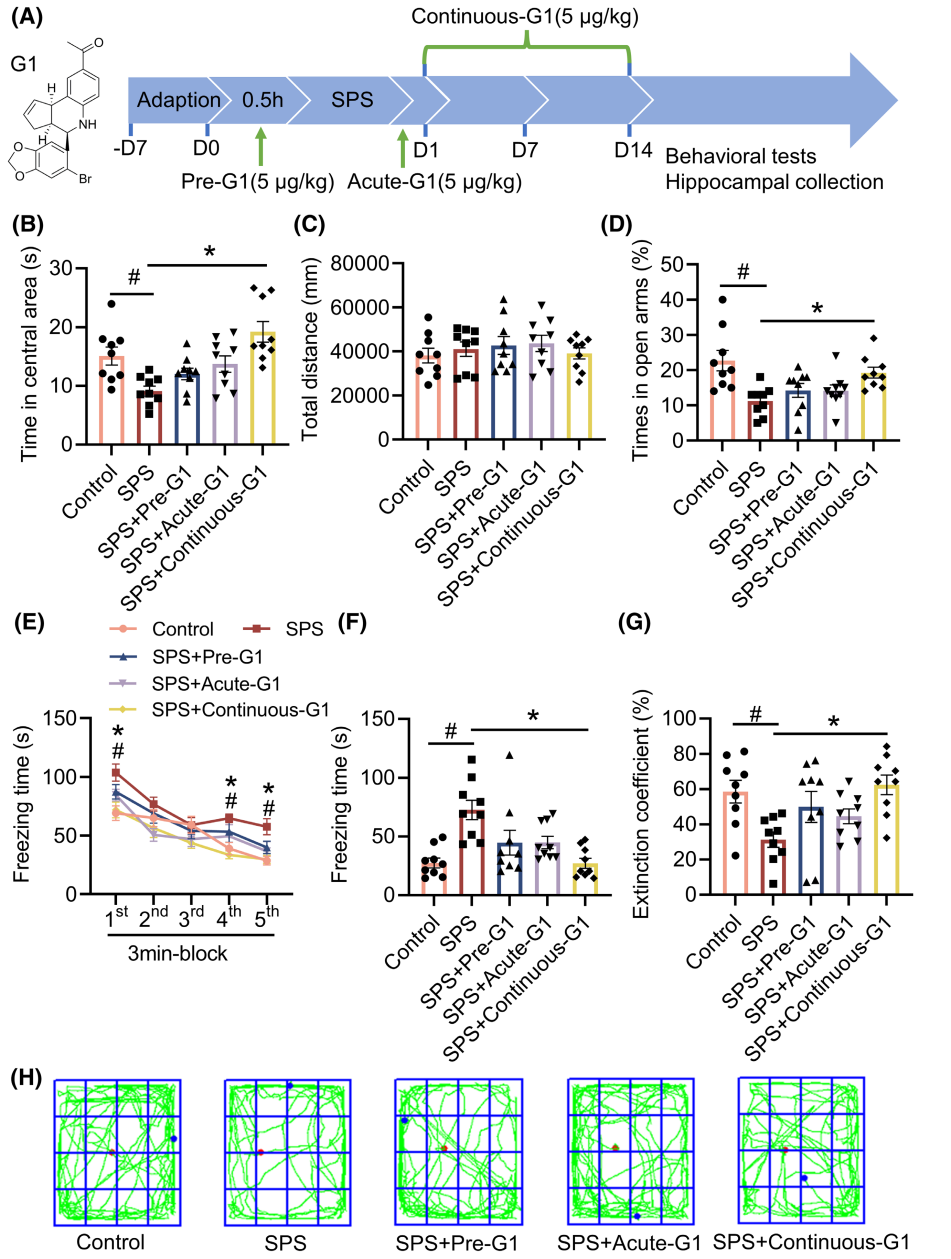
Continuous intraperitoneal injection of G1 improved post‐traumatic stress disorder (PTSD)‐like behaviors in single prolonged stress (SPS) mice. (A) Chemical structure of G1 and schematic illustration of the study design of *Experiment 3*; (B) movement time in central area of the open field test (OFT); (C) total distance in the OFT; (D) percentage of time spent exploring the open arms in the elevated plus‐maze test (EPMT); (E) freezing time during the re‐exposure phase of the fear conditioning task (FCT); (F) freezing time in the 24 h post‐fear test; (G) the extinction coefficient; (H) Total motion trajectory diagram for the OFT. Data are expressed as mean ± SEM (*n* = 9). ^#^ *p* < 0.05 versus the control group; *p < 0.05 versus the SPS group (Tukey's test or Dunn's test).

Considering the reduced expression of GPER1 in the hippocampus of SPS mice, the study administered intraperitoneal injections of the GPER1 activator G1 at three time points (Figure 2A). The results showed that in OFT, SPS mice spent significantly less time in the central region compared to the control group, while mice in the SPS + Continuous‐G1 group spent significantly more time in the central region (vs. SPS, *p* < 0.05, *F*[4, 40] = 7.788; Figure 2B). In addition, G1 administration did not affect the total distance, and there was no significant difference in the total distance among the groups, indicating that G1 administration did not affect the motor function of the mice (Figure 2C,H). Similarly, in EPMT, SPS mice had a significantly lower percentage of time spent in the open arm compared to the control group, whereas mice in the SPS + Continuous‐G1 group could significantly increase their time spent in the open arm (vs. SPS, *p* < 0.05, *F*[4, 40] = 5.446; Figure 2D).

In the re‐exposure period of FCT, the freezing time gradually decreased in all groups of mice, but in the later part of the re‐exposure period, SPS group still showed significantly higher freezing time compared to the control and SPS + Continuous‐G1 groups (time: *p* < 0.05, *F*[3.388, 135.5] = 54.16; groups: *p* < 0.05, *F*[4, 40] = 4.804; time × groups: *p* > 0.05, *F*[16, 160] = 1.421; Figure 2E). After 24 h of extinction, the freezing time of the SPS group was significantly higher than that in the control group; whereas, the freezing time of the SPS + Continuous‐G1 group was significantly lower (vs. SPS, *p* < 0.05, *F*[4, 40] = 7.056; Figure 2F). In addition, G1 administration could significantly increase the extinction coefficient in SPS mice (vs. SPS, *p* < 0.05, *F*[4, 40] = 4.078; Figure 2G). The results suggest that administering G1 continuously for 14 days leads to improvements in PTSD‐like behaviors in SPS mice. However, giving G1 30 min before and immediately after the modeling does not significantly impact SPS mice.

### 3.3 Continuous intraperitoneal injection of G1 inhibits abnormal hippocampal gamma oscillation in SPS mice

**FIGURE 3:**
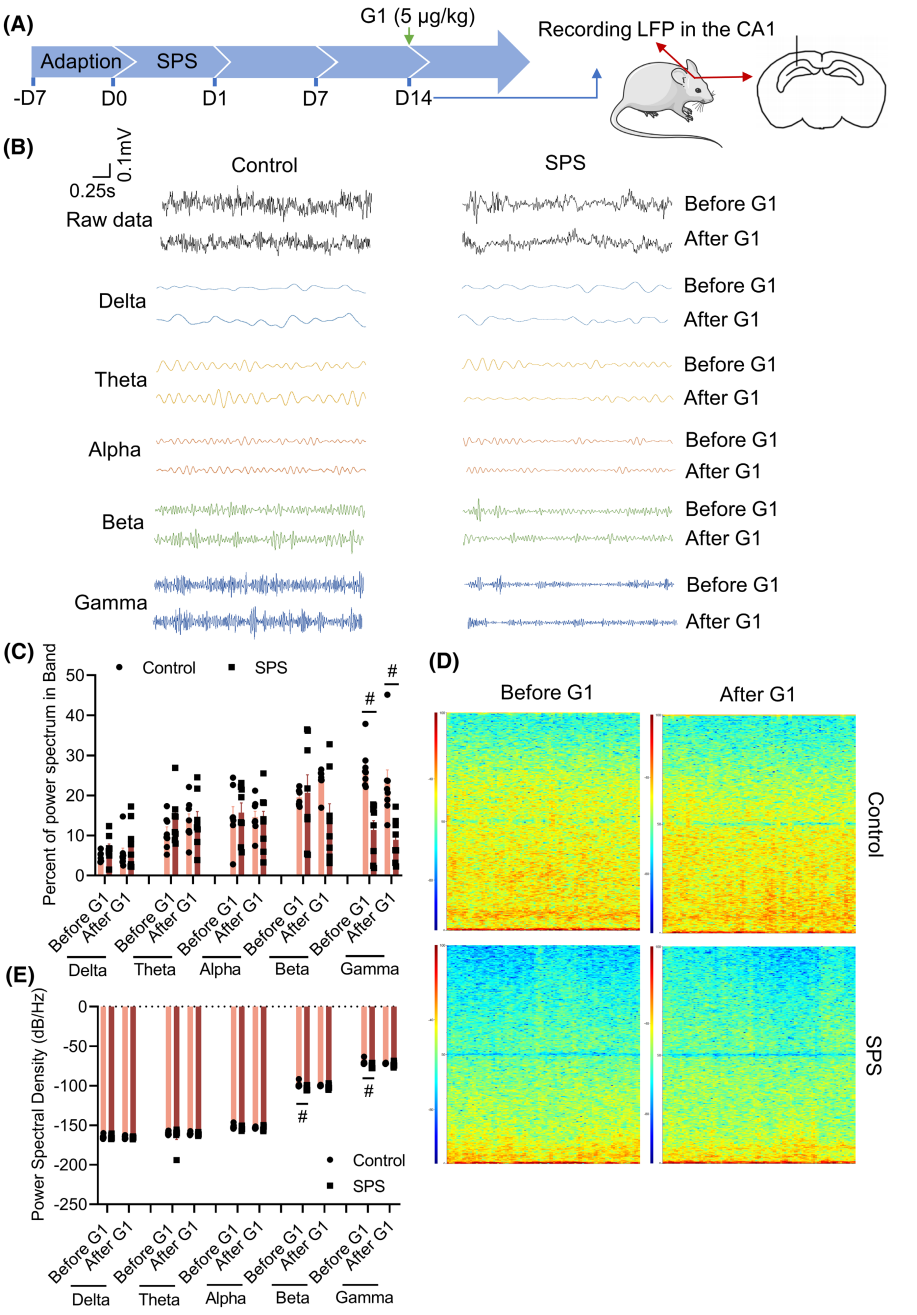
Acute G1 administration did not affect local field potential (LFP) in the hippocampal CA1 region of single prolonged stress (SPS) mice. (A) Schematic of G1 administration time; (B) representative diagrams of waveforms in each frequency band; (C) the percent power spectrum of different bands; (D) schematic of energy profile; (E) PSD. Data are expressed as mean ± SEM (*n* = 4). ^#^ *p* < 0.05 versus the control group (Bonferroni's test).

We observed LFP in the CA1 region of the hippocampus in mice from the control and SPS groups on day 14 after modeling. A single dose of G1 was administered to those mice 10 min later to assess the acute effects of G1on hippocampal oscillations on 14th day. We performed the percent power spectrum of different bands by dividing the LFP signal into five frequency bands according to a certain range and counting the ratio of each band to the overall signal. The results showed that before G1 administration, the ratio of gamma band in SPS group was significantly reduced (vs. control, *p* < 0.05), and the ratios of delta, theta, alpha, and beta bands were not significantly changed. After G1 administration, the gamma band of SPS group remained significantly lower (vs. control, *p* < 0.05; Figure 3A–C). At the same time, we compared the energy profiles of control and SPS group in 100 s. The results indicated that the energy content in the SPS group was significantly lower than that in the control group both before and after drug administration, especially in the gamma band (vs. control, *p* < 0.05; Figure 3D). In addition, we also counted the distribution of the power of the LFP signal in the frequency domain, i.e., PSD. It was found that before the administration of G1, the beta and gamma bands of SPS group were significantly lower (vs. control, *p* < 0.05), and there were no significant differences in Delta, Theta, and Alpha frequency bands. After the administration of G1, there were no significant changes in all frequency bands in the SPS group and control group (Figure 3E). These results indicate that acute G1 treatment at 14th day had no significant effect on the abnormal changes in LFP in the CA1 region of the hippocampus of SPS mice.

**FIGURE 4:**
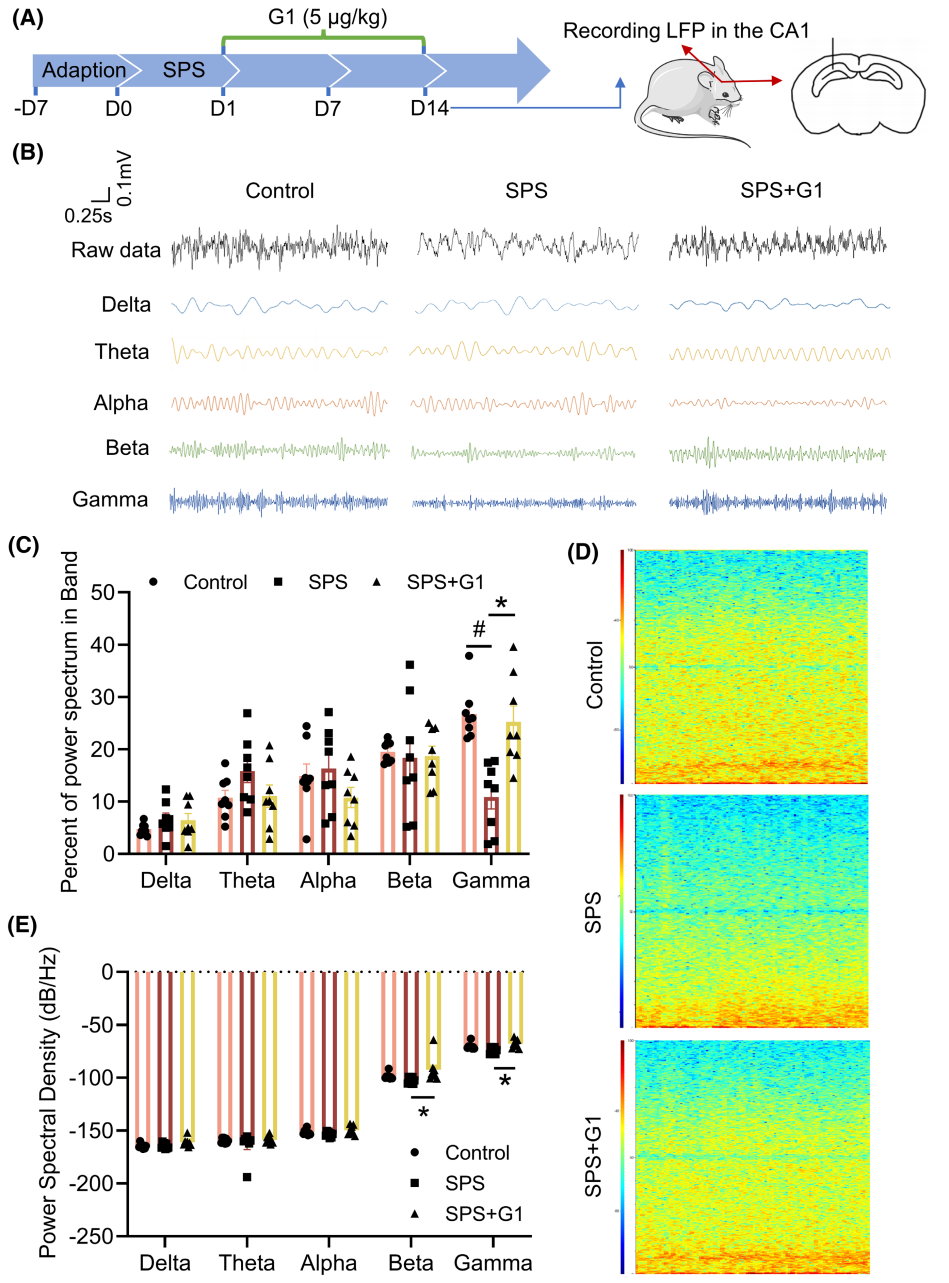
Abnormal changes in gamma band in single prolonged stress (SPS) mice were reversed by continuous administration of G1. (A) Schematic of G1 administration time; (B) representative diagrams of waveforms in each frequency band; (C) the percent power spectrum of different bands; (D) schematic of energy profile; (E) PSD. Data are expressed as mean ± SEM (*n* = 4). ^#^ *p* < 0.05 versus the control group; *p < 0.05 versus the SPS group (Tukey's test).

At the same time, we monitored LFP in the CA1 region of the hippocampus of mice in the group administered for 14 consecutive days. The results of the percent power spectrum of different bands showed that the ratio of the gamma band was significantly higher after G1 administration (vs. SPS, *p* < 0.05; Figure 4A–C). The energy profiles results revealed that the G1 group contains significantly higher energy compared to the SPS group, especially in the gamma band (Figure 4D). In addition, the PSD results showed that there were no significant changes in all frequency bands in SPS group compared with the control group, whereas G1 treatment given for 14 consecutive days significantly elevated the beta and gamma bands in SPS group (vs. SPS, *p* < 0.05; Figure 4E). Taken together, the results showed that G1 administration for 14 consecutive days significantly suppress the abnormal changes in the gamma band in the CA1 region of the hippocampus of SPS mice.

### 3.4 G1 attenuates synaptic damage and abnormal mitochondrial protein expression in the hippocampus of SPS mice

**FIGURE 5:**
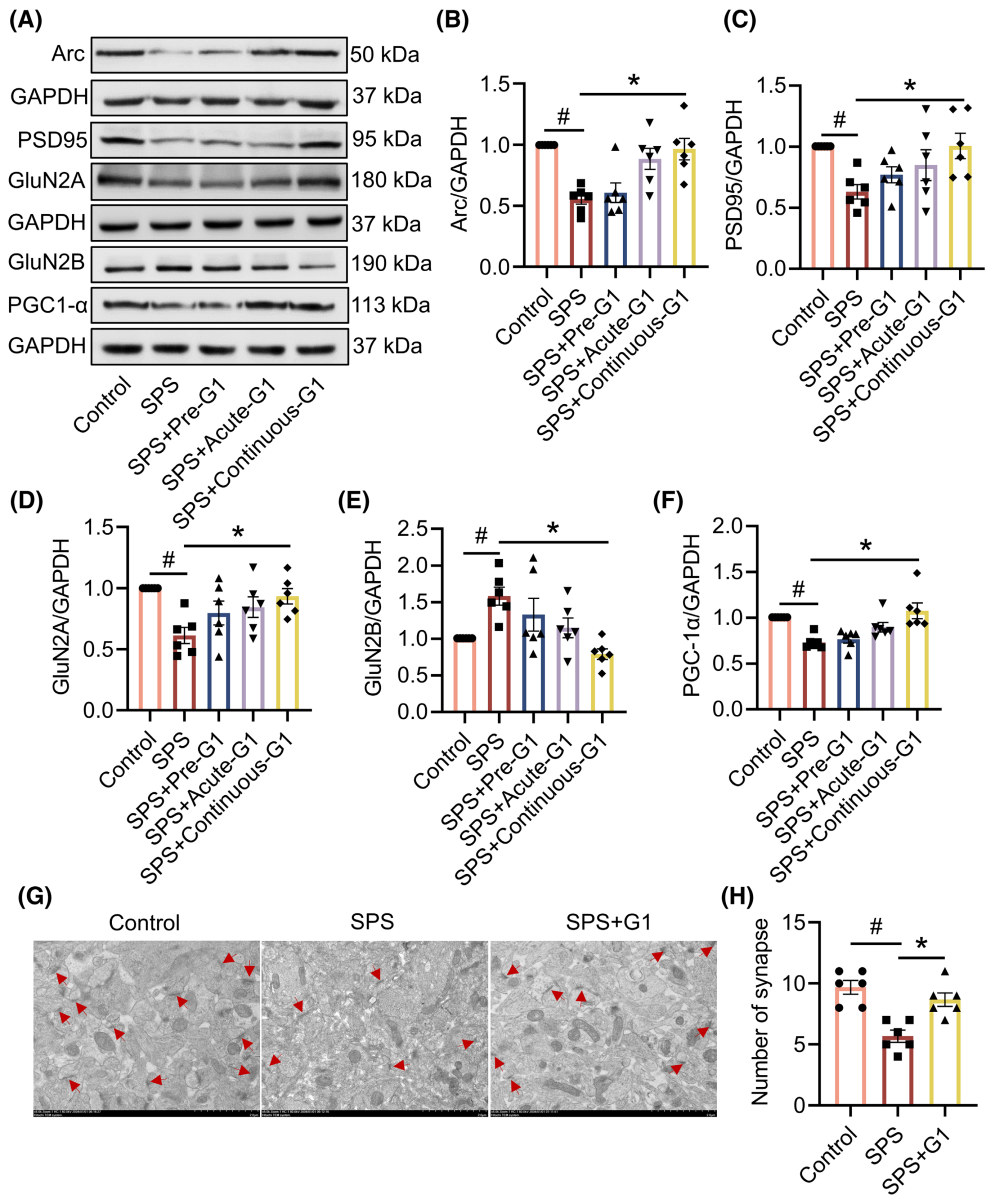
G1‐attenuated synaptic damage and aberrant mitochondrial protein expression in the hippocampus of single prolonged stress (SPS) mice. (A) Representative blots of Arc, PSD95, GluN2A, GluN2B, and PGC‐1α (*n* = 3); (B) quantitative data of Arc; (C) quantitative data of PSD95; (D) quantitative data of GluN2A; (E) quantitative data of GluN2B; (F) quantitative data of PGC‐1α; (G) schematic diagram of synaptic structures in the CA1 region; arrows indicate synaptic structures (*n* = 3); (H) number of synapses. Data are expressed as mean ± SEM. ^#^ *p* < 0.05 versus the control group; *p < 0.05 versus the SPS group. (Tukey's test or Dunn's test).

We detected synapse‐ and mitochondria‐associated proteins by Western blot. The results revealed that the expression levels of Arc, PSD95, and GluN2A proteins were reduced, whereas GluN2B expression was elevated in the hippocampus of mice in the SPS group compared to the control group. Following 14 consecutive days of G1 treatment, there was a significant increase in the expression levels of Arc (vs. SPS, *p* < 0.05, *H* = 17.87), PSD95 (vs. SPS, *p* < 0.05, *F*[4, 25] = 3.638), and GluN2A (vs. SPS, *p* < 0.05, *F*[4, 25] = 4.378), along with a significant decrease in GluN2B (vs. SPS, *p* < 0.05, *F*[4, 25] = 5.261; Figure 5A–E). Meanwhile, PGC‐1αwas decreased in the hippocampus of SPS mice, whereas its expression was significantly increased after G1 administration for 14 consecutive days (vs. SPS, *p* < 0.05, *H* = 21.21; Figure 5F).

In addition, we used transmission electron microscopy to observe the effects of continuous 14‐day administration on synapses in the CA1 region of the hippocampus. The results showed that the number of synapses was significantly reduced in SPS mice compared to the control group, and the number of synapses was significantly increased after G1 administration (vs. SPS, *p* < 0.05, *F*[2, 15] = 15.00; Figure 5G,H).

### 3.5 G1 increases GPER1 expression and promotes BDNF/TrkB signaling in SPS mice

**FIGURE 6:**
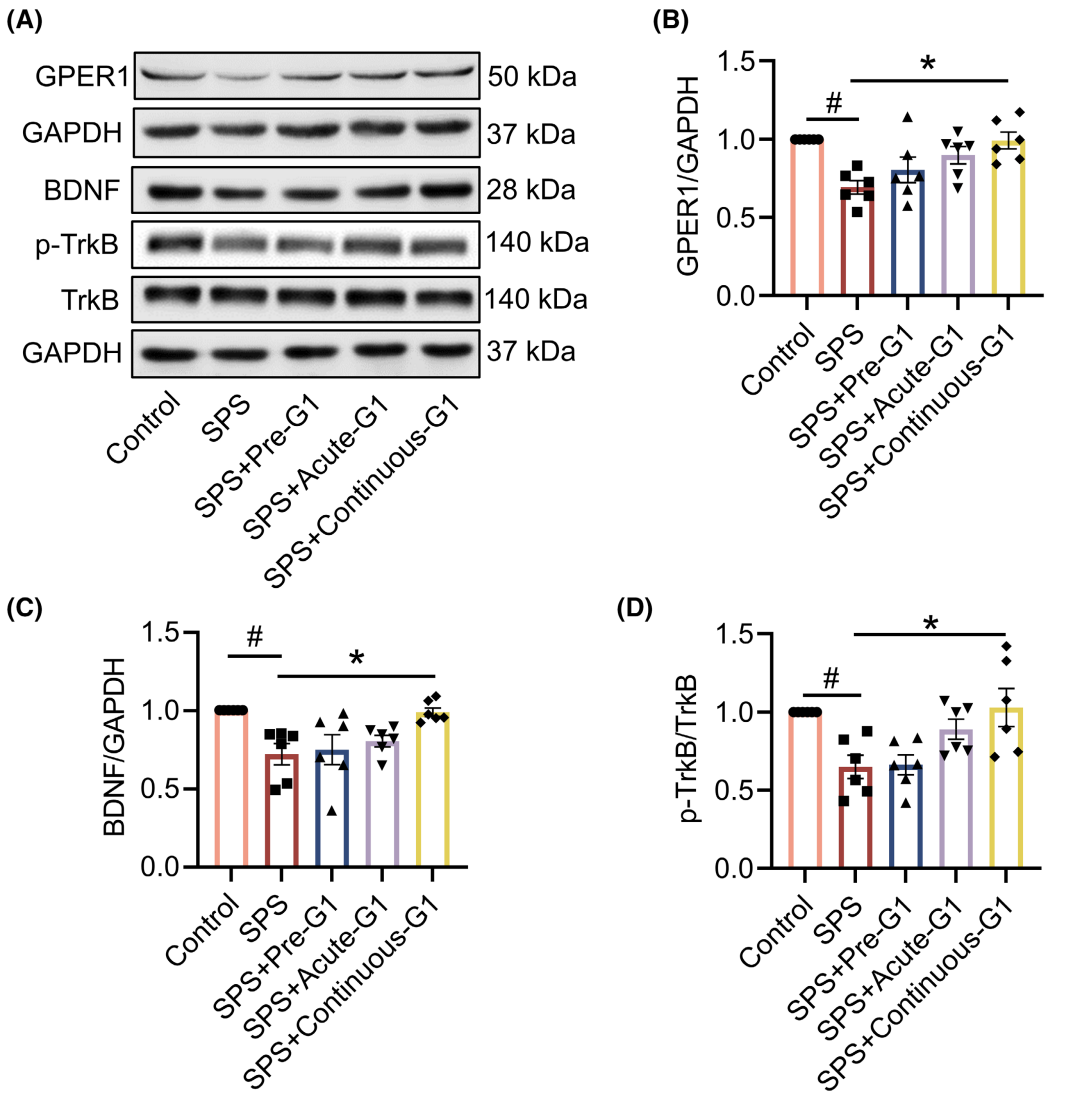
G1 increased G protein‐coupled estrogen receptor 1 (GPER1) expression and promoted BDNF/TrkB signaling in single prolonged stress (SPS) mice. (A) Representative blots of GPER1, BDNF, p‐TrkB, and TrkB; (B) quantitative data of GPER1; (C) quantitative data of BDNF; (D) quantitative data of p‐TrkB and TrkB. Data are expressed as mean ± SEM (*n* = 3). ^#^ *p* < 0.05 versus the control group; *p < 0.05 versus the SPS group (Tukey's test).

Subsequently, we tested whether G1 could increase the expression of GPER1 and also whether G1 had an ameliorative effect on the abnormal BDNF/TrkB signaling in SPS mice. The results showed that GPER1 expression was decreased in the hippocampus of SPS group compared with the control group, whereas GPER1 expression was significantly increased after administration of G1 for 14 consecutive days (vs. SPS, *p* < 0.05, *F*[4, 25] = 5.889; Figure 6A,B). Moreover, hippocampal BDNF (vs. SPS, *p* < 0.05, *H* = 19.91) and p‐TrkB (vs. SPS, *p* < 0.05, *F*[4, 25] = 5.828) were significantly reduced in the SPS group of mice, which was reversed by the administration of G1 treatment for 14 consecutive days (Figure 6C,D). This result indicates that G1 treatment given for 14 consecutive days increases the expression of GPER1 and promotes the expression of the BDNF/TrkB signaling in SPS mice.

### 3.6 The TrkB inhibitor ANA‐12 as well as GPER1 antagonist G15 blocks the ameliorative effects of G1 on PTSD‐like behaviors

**FIGURE 7:**
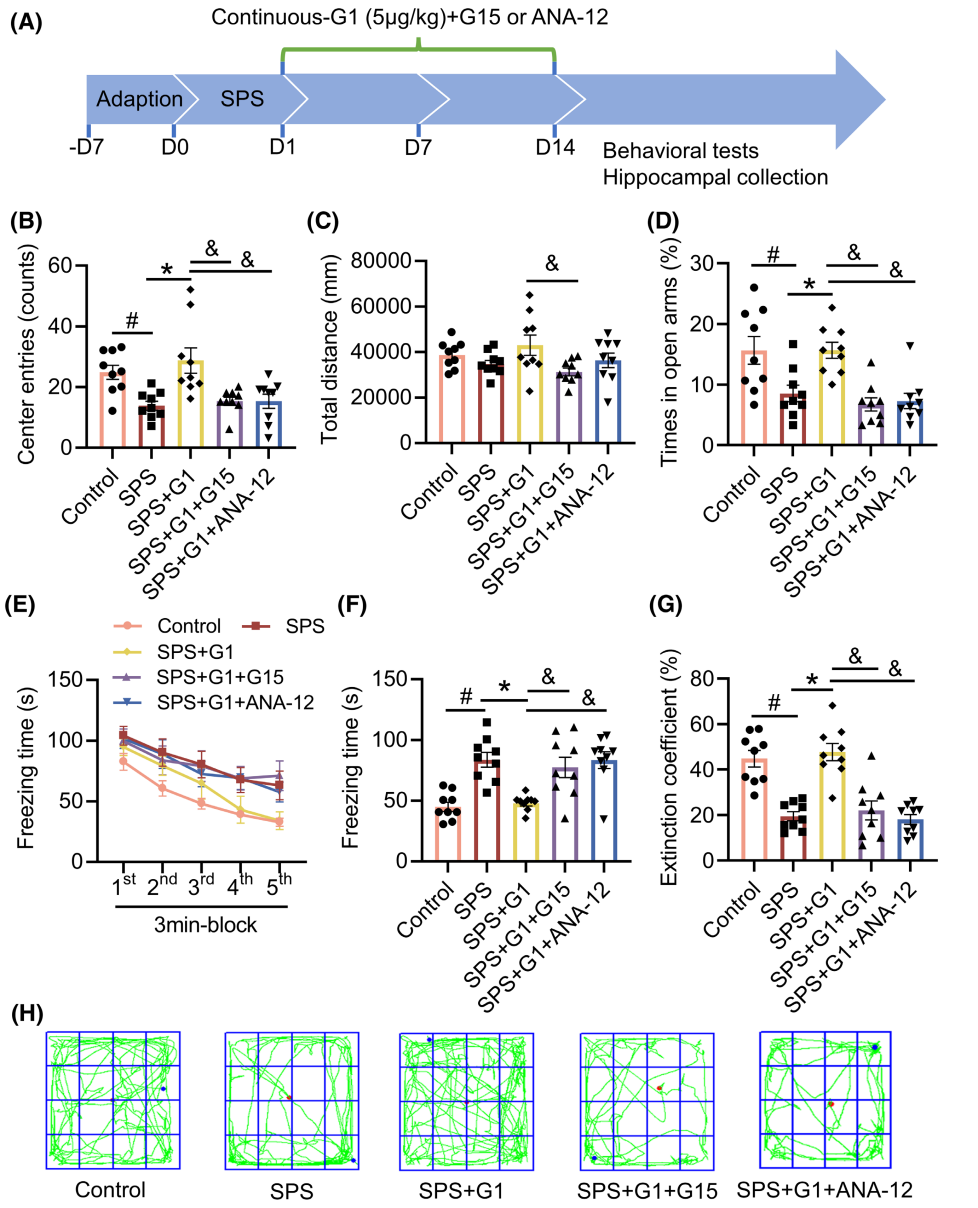
Improvement of PTSD‐like behaviors by G1 was blocked by G15 and ANA‐12. (A) Schematic illustration of the study design of *Experiment 5*; (B) total number of entries in the central area in the open field test (OFT); (C) total distance in the OFT; (D) percentage of time spent exploring the open arms in the elevated plus‐maze test (EPMT); (E) freezing time during the re‐exposure phase of the fear conditioning task (FCT); (F) freezing time in the 24‐h post‐fear test; (G) the extinction coefficient; (H) total motion trajectory diagram for the OFT. Data are expressed as mean ± SEM (*n* = 9). ^#^ *p* < 0.05 versus the control group; *p < 0.05 versus the SPS group; ^&^ *p*<0.05 versus the SPS + G1 group(Tukey's test or Dunn's test).

To demonstrate the role of BDNF/TrkB signaling in the protective effects of G1 against PTSD, we administered G1 in combination with the TrkB inhibitor ANA‐12. Additionally, we combined G1 with G15, a specific GPER1 inhibitor, to further validate our findings (Figure 7A). As shown in Figure 7B, in OFT, the number of mice crossing the central region was significantly lower in the SPS + G1 + G15 and SPS + G1 + ANA‐12 groups (vs. SPS + G1, *p* < 0.05, *H* = 21.21). Meanwhile, ANA‐12 did not affect the total distance, whereas G15 administration significantly decreased the total distance (vs. control, *p* < 0.05, *F*[4, 40] = 2.475; Figure 7C,E). In EPMT, the percentage of time spent in the open arm was also significantly lower in the SPS + G1 + G15 and SPS + G1 + ANA‐12 groups (vs. SPS + G1, *p* < 0.05, *H* = 20.91; Figure 7D).

In the re‐exposure period of FCT, the freezing time gradually decreased over time in all groups, but in the later part of the re‐exposure period, the freezing time remained high in the SPS + G1 + G15 and SPS + G1 + ANA‐12 groups (vs. SPS + G1, time: *p* < 0.05, *F*[3.571, 142.8] = 49.05; groups: *p* > 0.05, *F*[4, 40] = 2.422; time × groups: *p* > 0.05, *F*[16, 160] = 0.9228; Figure 7E). After 24 h of extinction, the freezing time was still significantly higher in the SPS + G1 + G15 and SPS + G1 + ANA‐12 groups (vs. SPS + G1, *p* < 0.05, *H* = 22.79; Figure 7F). Meanwhile, both G15 and ANA‐12 significantly reduced the extinction coefficient in SPS mice (vs. SPS + G1, *p* < 0.05, *F*[4, 40] = 19.70; Figure 7G). These results indicated that ANA‐12 functions similarly to G15, inhibiting the ameliorative effects of G1 on PTSD‐like behaviors in SPS mice.

### 3.7 ANA‐12 as well as G15 blocks the improvement effect of G1 on abnormal synaptic protein expression in SPS mice

**FIGURE 8:**
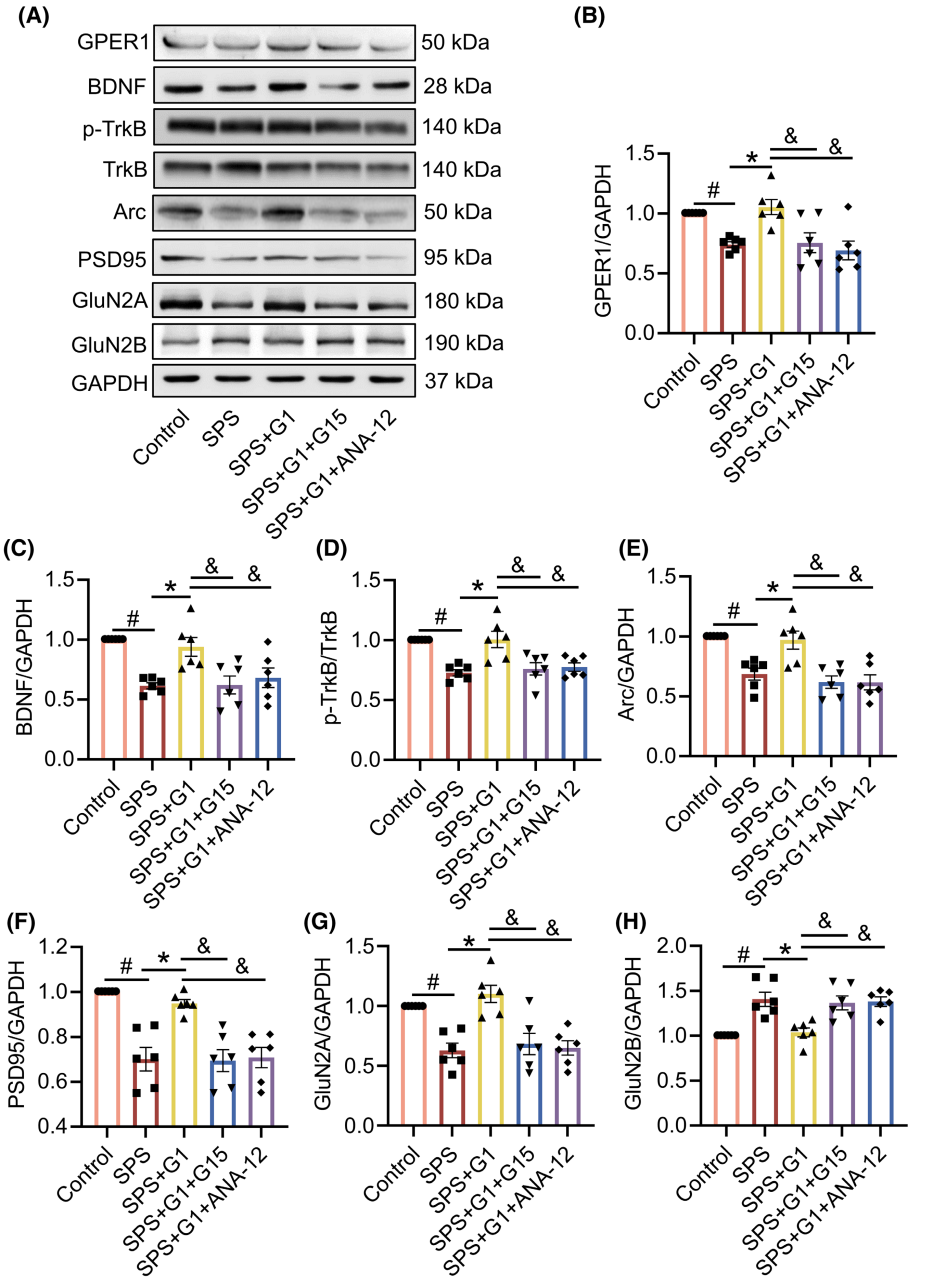
G15 and ANA‐12 blocked the ameliorative effect of G1 on the aberrant expression of synaptic proteins in the hippocampus of single prolonged stress (SPS) mice. (A) Representative blots of GPER1, BDNF, p‐TrkB, TrkB, Arc, PSD95, GluN2A, and GluN2B; (B) quantitative data of GPER1; (C) quantitative data of BDNF; (D) quantitative data of p‐TrkB and TrkB; (E) quantitative data of Arc; (F) quantitative data of PSD95; (G) quantitative data of GluN2A; (H) quantitative data of GluN2B. Data are expressed as mean ± SEM (*n* = 3). ^#^ *p* < 0.05 versus the control group; *p < 0.05 versus the SPS group; ^&^ *p*<0.05 versus the SPS + G1 group (Tukey's test).

We used Western blot to examine the effects of G15 and ANA‐12 on synapse‐related proteins in SPS mice. The results showed that ANA‐12 had a similar effect as G15, and both could inhibit the increase in the expression levels of GPER1 (vs. SPS + G1, *p* < 0.05, *F*[4, 25] = 7.884; Figure 8A,B), BDNF (vs. SPS + G1, *p* < 0.05, *F*[4, 25] = 8.971; Figure 8C), p‐TrkB (vs. SPS + G1, *p* < 0.05, *F*[4, 25] = 10.08; Figure 8D), Arc (vs. SPS + G1, *p* < 0.05, *F*[4, 25] = 12.24; Figure 8E), PSD95 (vs. SPS + G1, *p* < 0.05, *F*[4, 25] = 15.26; Figure 8F) and GluN2A (vs. SPS + G1, *p* < 0.05, *F*[4, 25] = 12.03; Figure 8G), and to increase the expression level of GluN2B (vs. SPS + G1, *p* < 0.05, *F*[4, 25] = 11.54; Figure 8H) in the hippocampus of the SPS mice after the administration of G1.

### 3.8 ANA‐12 as well as G15 blocks G1‐mediated mitochondrial damage repair in SPS mice

**FIGURE 9:**
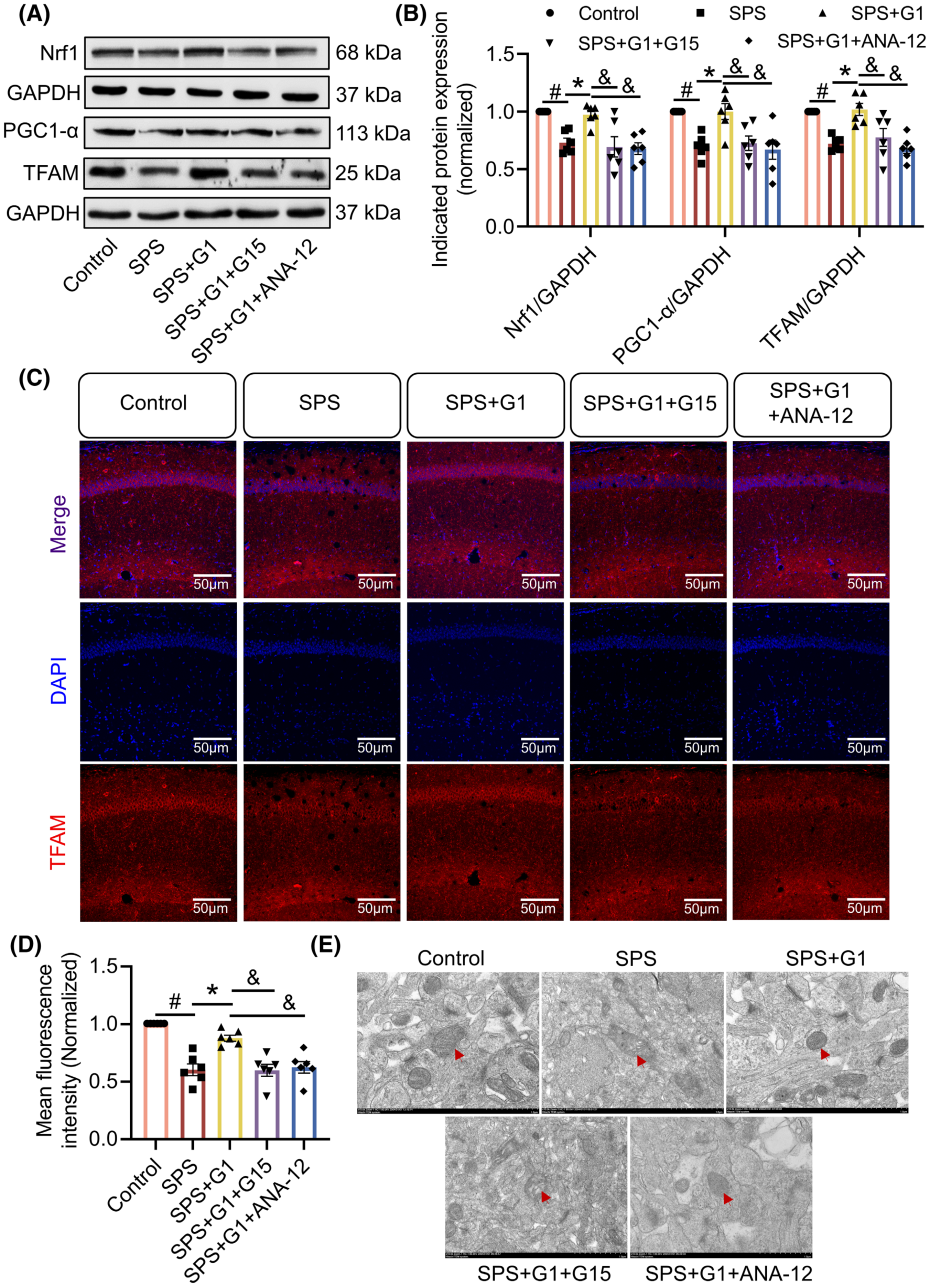
Amelioration of mitochondrial damage by G1 was blocked byG15 and ANA‐12. (A) Representative blots of Nrf1, PGC1‐α, and TFAM (*n* = 3); (B) quantitative data of Nrf1, PGC1‐α, and TFAM; (C) typical immunofluorescence images showing TFAM expression in the CA1 region (*n* = 3); (D) mean fluorescence intensity of TFAM in the CA1 region; (E) schematic representation of the mitochondrial structure of mice hippocampal CA1 region, arrows indicate mitochondria(*n* = 3). Data are expressed as mean ± SEM. ^#^ *p* < 0.05 versus the control group; *p < 0.05 versus the SPS group; ^&^ *p* < 0.05 versus the SPS + G1 group(Tukey's test).

We used Western blot to detect the expression levels of the mitochondria‐related proteins PGC1‐α, Nrf1, and TFAM. The results revealed that the protein expression levels of PGC1‐α (vs. SPS + G1, *p* < 0.05, *F*[4, 25] = 8.016), Nrf1 (vs. SPS + G1, *p* < 0.05, *F*[4, 25] = 9.536) and TFAM (vs. SPS + G1, *p* < 0.05, *F*[4, 25] = 11.46) in the hippocampus of SPS mice were significantly decreased, and their expression levels increased significantly after G1 administration, while both G15 and ANA‐12 reversed these changes (Figure 9A,B). Subsequently, we examined the expression of TFAM in the hippocampus using immunofluorescence staining. The results demonstrated that the mean fluorescence intensity of TFAM in the hippocampal CA1 region of SPS mice was markedly diminished. This reduction was significantly reversed by G1 administration, but subsequently, there was a notable decrease in TFAM expression following the administration of G15 and ANA‐12 (vs. SPS + G1, *p* < 0.05, *F*[4, 25] = 20.38; Figure 9C,D). We also used transmission electron microscopy to observe the changes in mitochondrial morphology. The results showed that hippocampal mitochondria in SPS mice were damaged with broken ridges, lost their structure, and appeared to be vacuolated when compared with the control mice, and that G1 was effective in ameliorating those changes. In addition, G15 and ANA‐12 could inhibit the effects of G1, respectively (Figure 9E).

## 4 DISCUSSION

**FIGURE 10:**
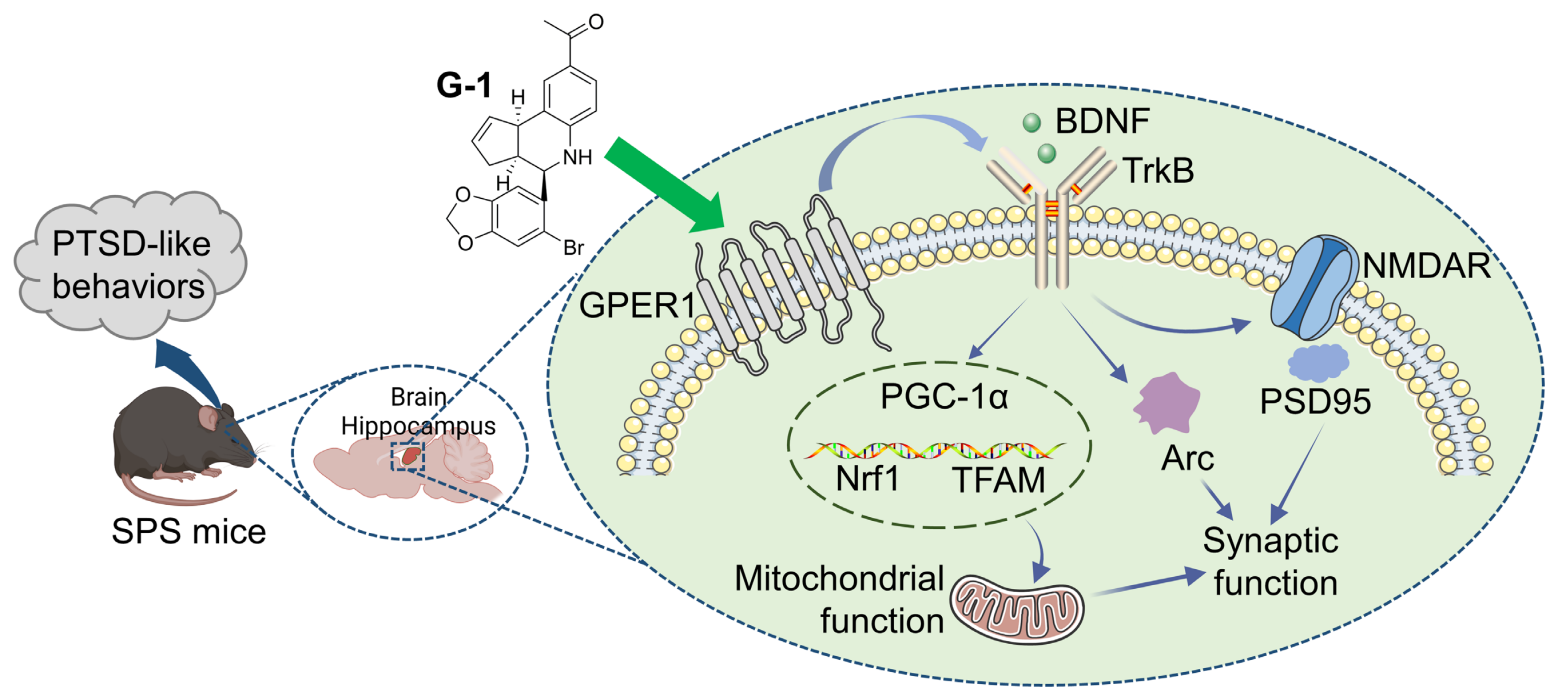
The mechanism of the G protein‐coupled estrogen receptor 1 (GPER1) agonist G1 in improving post‐traumatic stress disorder (PTSD)‐like behaviors for SPS mice.G1 improved PTSD‐like behaviors in SPS mice, possibly by increasing hippocampal GPER1 expression and promoting BDNF/TrkB signaling to repair synaptic and mitochondrial functional impairments.

In this study, we identified the changes of GPER1 in the hippocampus of SPS mice and revealed the efficacy and mechanism of action of the GPER1 agonist G1 in improving PTSD‐like behaviors (Figure 10). Our findings suggested that GPER1 expression was reduced in SPS mice, whereas G1 promoted GPER1 expression and improved PTSD‐like behaviors. We demonstrated that G1 administration repaired hippocampal synaptic and mitochondrial functional impairments in SPS mice and suppressed abnormal changes in the gamma band in the CA1 region of the hippocampus. Additionally, we used the TrkB inhibitor and GPER1 antagonist to verify that BDNF/TrkB was directly involved in the ameliorative process of G1in SPS mice.

### 4.1 GPER1 may be a potential target for the treatment of PTSD

In recent years, more and more studies have found that estrogen plays a very important role in the prevalence of PTSD.^9^, ^33^ Estrogen is a steroid hormone that has multiple physiological functions and acts through estrogen receptor signaling.^34^, ^35^ Activation of GPER1, a novel membrane receptor for estrogen, has important roles in biological processes, such as improving learning memory and anxiety or depression behaviors in mice.^36^, ^37^, ^38^ Our previous study also found that GPER1 activation repaired fear memory impairment in aging mice.^31^ Fear memory abnormalities are also a core feature of PTSD, yet there are no reports on the effect of GPER1 on PTSD. In addition, another study has shown that there is no significant difference in the distribution of GPER1 between adult males and females.^39^ However, estrogen secreted by the reproductive system of females is cyclical and GPER1 expression is influenced by serum estrogen levels.^40^ Thus, to exclude the effect of estrogen on GPER1, studies have usually used males or de‐ovulated females. In this study, we first examined the dynamics of GPER1expressionin SPS mice using male mice, and our results showed that GPER1 declined at the 7th day after modeling of SPS mice, and was significantly reduced at the 14th day. Then, we used OVX female mice to again examine the changes in GPER1after SPS modeling and showed that GPER1 expression was similarly reduced at 14th day, aligning with the outcomes observed in male mice. In addition, we showed that GPER1 was mainly distributed on neurons and glial cells, which was consistent with the results of previous studies.^41^ A study has investigated the effects of GPER1 knockdown on anxiety‐like behavior in rats and found that GPER1 knockdown resulted in anxiety‐like behavior, decreased serum corticosterone levels, and found that anxiety‐like behavior was exacerbated by SPS stimulation.^42^ Therefore, we speculated that there might be a relationship between the PTSD‐like behaviors and the decreased expression of GPER1 and that the decreased expression may be related to the fluctuation of hormone levels after experiencing trauma.

### 4.2 G1 ameliorates PTSD‐like behaviors by repairing synaptic and mitochondrial functional impairments

The core symptoms of PTSD are characterized by abnormal fear memory accompanied by anxious or depressive behaviors. We have not found direct research linking GPER1 to PTSD, but emerging evidence suggests that estrogen signaling plays a critical role in modulating stress responses and emotional regulation.^43^ Interestingly, similar studies have demonstrated that G1 could alleviate anxiety‐like behaviors^37^, ^44^, ^45^ or abnormal fear memory in animals by activating GPER1 and subsequently triggering downstream pathways.^31^ Our results showed that 14 consecutive days of G1 administration were effective in improving the anxiety‐like behavior and fear memory abnormalities in SPS mice.

Subsequently, we applied LFP to record whether G1 administration affected neural oscillations in the CA1 region of the hippocampus. It is suggested that the efficacy of a single acute dose may not be sufficient to prompt the action of GPER1 and that successive administrations may be required to produce a cumulative effect and thus exert its function. Moreover, gamma oscillations are mainly associated with functions such as memory and cognition, and their reduction indicates impaired memory and cognition in SPS mice.^46^ Meanwhile, it was found that anxiety levels of anxious patients were negatively correlated with gamma band, which is consistent with our experimental results.^47^ At the same time, we also found that continuous G1 administration could reverse the abnormal changes in the gamma band, which is consistent with the results that G1 could improve PTSD‐like behaviors in SPS mice.

In addition, we found that hippocampal GPER1 expression in SPS mice was significantly increased after continuous intraperitoneal injection of G1, butG1 administration before and immediately after modeling did not increase GPER1 expression. We speculate that there may be several reasons for this: First, continuous G1 treatment provides sufficient and sustained stimulation to activate signaling pathways, leading to increased transcription and protein synthesis ofGPER1in the hippocampus of SPS mice. Second, cells may adapt to persistent G1 stimuli by modulating receptor expression in response to environmental changes. Continuous G1 treatment may also induce an adaptive response in cells, enhancing their sensitivity and responsiveness to estrogen signals by upregulating GPER1 expression. We have previously conducted sufficient research on the impairment of synaptic function in SPS mice and have found that hippocampal synaptic function is an important player in the regulation of fear memory abnormalities in PTSD,^22^ and the normal expression of synaptic plasticity proteins plays an important role in the execution of synaptic functions. Consistent with previous findings, we found that PSD95, Arc, and GluN2A protein expression was reduced and GluN2B protein expression was elevated in the hippocampus of SPS mice, indicating that synaptic function was impaired in SPS mice, which could be reversed by continuous G1 treatment.

Moreover, recent studies have found that impaired mitochondrial function is also a key factor in the development of PTSD, as well as an association between impaired mitochondrial function and chronic stress response and emotional instability.^48^, ^49^ We found that the repair of mitochondrial function was effective in ameliorating the abnormalities of fear memory, and PGC1‐α was a key target in this process.^27^ PGC1‐α is a key mediator of mitochondrial energy metabolism and plays a role in regulating mitochondrial function.^50^ Nrf1 is a mitochondrial transcription factor and a redox determinant indispensable for the maintenance of mitochondrial homeostasis.^51^ TFAM, as a transcription factor and one of the downstream regulators of PGC1‐α, is regulated by PGC1‐α together with Nrf1.^52^ Our findings revealed a reduced expression of PGC1‐α, Nrf1, and TFAM in the hippocampus of SPS mice, suggesting dysfunction of mitochondrial energy metabolism. Disturbed mitochondrial energy metabolism could lead to abnormalities in neuronal functions such as action potential and neurotransmitter release.^53^ After G1 treatment, the expression of PGC1‐α, Nrf1, and TFAM rebounded, and the results of transmission electron microscopy also showed that G1 was effective in ameliorating the damage to the morphology and structure of mitochondria in SPS mice. These results suggest that the improvement of the PTSD‐like behaviors by G1 may be related to the repair of mitochondrial function.

However, synapses and mitochondria are not two completely separate entities. Mitochondria, as the central hub for the regulation of synapses, and the dynamic changes in their morphology and distribution along neuronal branches are crucial for synaptic functions.^54^ There is accumulating evidence that altered mitochondrial morphology or function leads to abnormal synaptic development, plasticity, and their functions.^55^, ^56^ At the same time, mitochondrial dysfunction leads to abnormal synaptic plasticity, causing exacerbation of PTSD symptoms.^57^ It has also been shown that chronic immobilization stress leads to oxidative damage to mitochondrial function and membrane lipids, resulting in abnormal neurotransmitter signaling in synapses and circuits, and that this abnormal neurotransmitter signaling may create the negative feedback that facilitates the pathophysiological process of PTSD, depression, or suicidal ideation.^58^ PGC1‐α could also affect synaptic transmission and plasticity in the hippocampus and is a key factor in synaptic transmission.^59^ Our previous findings revealed that activation of PGC1‐α reversed the aberrant expression of synapse‐related proteins in the hippocampus of SPS mice.^27^ Consequently, our findings indicated that G1 could improve PTSD‐like behaviors by repairing hippocampal synaptic and mitochondrial function.

### 4.3 BDNF/TrkB signaling involves in G1‐mediated repair of synapses and mitochondrial function

TrkB is a specific receptor for BDNF, and when BDNF binds to it, it can induce TrkB coupling and phosphorylation, forming the BDNF/TrkB signaling, which is involved in the regulation of synaptic plasticity and neuronal survival.^60^, ^61^ Previous study found that inhibition of the BDNF/TrkB signaling promotes PTSD‐like behaviors in SPS mice.^20^ Consistently, we found that continuous administration of G1 significantly increased the expression levels of BDNF and p‐TrkB in the hippocampus. Further results showed that the ameliorative effect of G1 on PTSD‐like behaviors could be inhibited by ANA‐12. We also used G15, a specific inhibitor of GPER1, as a comparison with ANA‐12, and found that the inhibitory effect of ANA‐12 on G1 was comparable to that of G15. This suggests that G1 could ameliorate PTSD‐like behaviors by activating the BDNF/TrkB signaling.

The involvement of the BDNF/TrkB signaling in the repair of synaptic function to ameliorate PTSD‐like behaviors has been well‐studied by our team.^20^, ^21^ BDNF has been reported to stimulate mitochondrial metabolism by increasing the efficiency of respiratory coupling and ATP synthesis.^62^ It has also been found that BDNF specifically regulates mitochondrial transport and induces more mitochondrial docking at presynaptic sites.^63^ PGC1‐α is a regulator of BDNF, and the pathway formed by those two mediates mitochondrial biogenesis and dynamics plays an important role in hippocampal synapse formation and maintenance.^64^ Activation of the BDNF/TrkB signaling could also increase PGC1‐α expression in neurons.^65^ We have previously found that PTSD‐like behaviors can be improved by promoting PGC1‐α expression to promote synaptic deficit.^27^ We therefore suggest that BDNF/TrkB activation can further increase PGC1‐α expression thereby contributing to mitochondrial biogenesis and increasing synaptogenesis.

### 4.4 Research limitations

Our study suggests that G1 may be a potential drug for the treatment of PTSD. However, our study leaves much to be solved. Firstly, we evaluated the efficacy of G1 in SPS mice and also observed that G1 had no effect on either anxiety‐like behavior or the extinction of fear memory in normal unstimulated mice. However, we found that GPER1 inhibitor, G15, suppressed the total distance of SPS mice, and speculated that the motor function of SPS mice might have been affected by the administration of G15 for 14 consecutive days. Meanwhile, we found that G15 did not affect the total locomotor distance of normal mice (Figure S1). Secondly, the function of GPER1 in specific cells should be distinguished.

## 5 CONCLUSION

In summary, G1 improved PTSD‐like behaviors in SPS mice, possibly by increasing hippocampal GPER1 expression and promoting BDNF/TrkB signaling to repair synaptic and mitochondrial functional impairments. This study warrants G1 as a candidate drug for the therapy of PTSD.

## AUTHOR CONTRIBUTIONS

L.C. and Y.Z. contributed equally to this work, completed experimental research, data analysis, and original draft. Z.W., Z.Z., and J.W. gave guidance on the experimental methods. G.Z. and S.Y designed the experiment and reviewed and edited the manuscript.

## CONFLICT OF INTEREST STATEMENT

None.

## Supporting information


Figure S1.

## Data Availability

The data that support the findings of this study are available from the corresponding author upon reasonable request.
