# Supplementary material for: Activation of GPER1 by G1 prevents PTSD‐like behaviors in mice: Illustrating the mechanisms from BDNF/TrkB to mitochondria and synaptic connection
Source: CNS Neurosci Ther. 2024 Jul 11;30(7):e14855. doi: 10.1111/cns.14855 (PMC11239537; doi:10.1111/cns.14855)
Supplement: Supplementary file 1 — Figure S1. [file CNS-30-e14855-s001.docx]

**sFig.1**


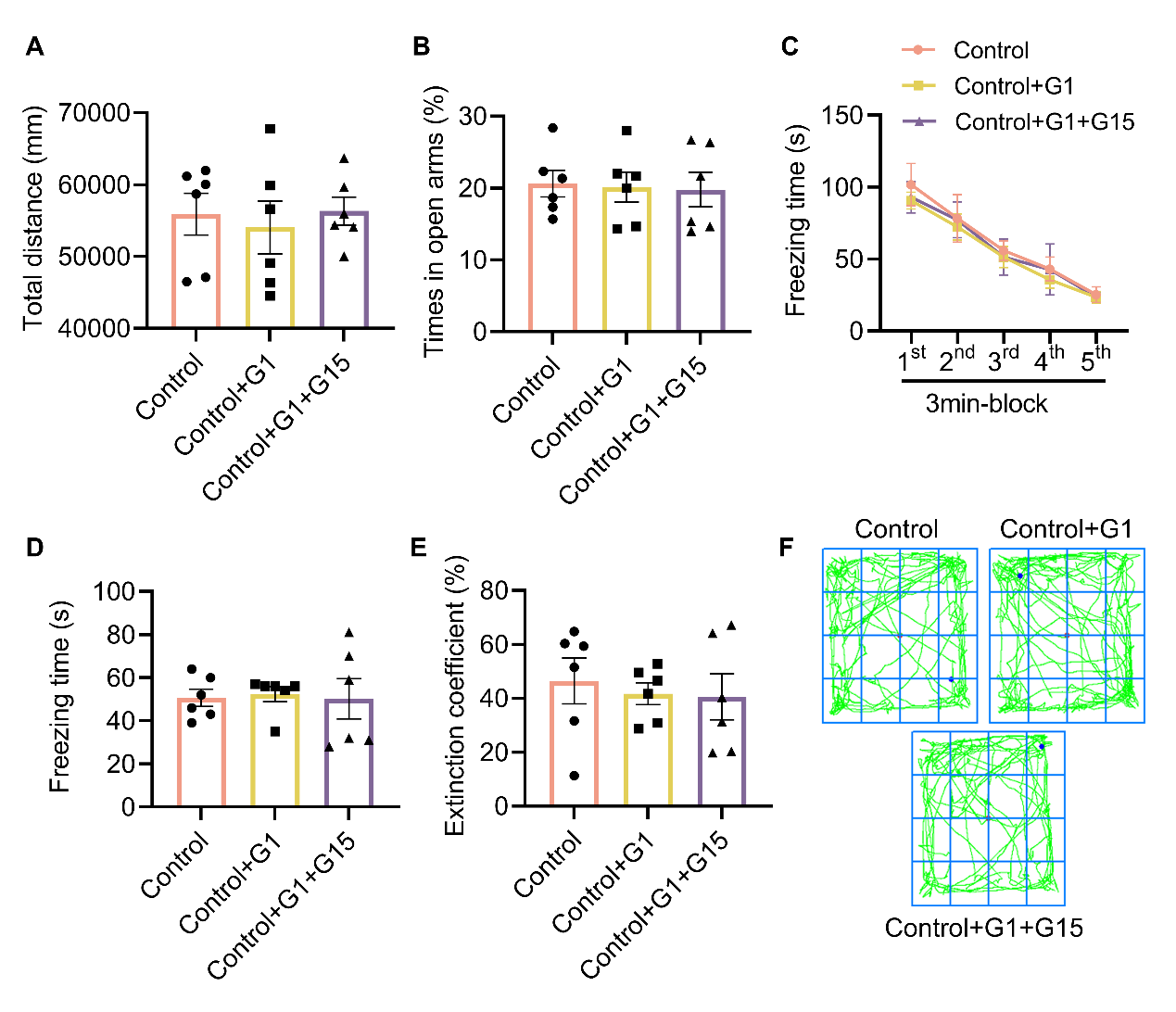


**sFig.1 G1 and G15 are ineffective on Control mice.** (A) Total distance in the OFT; (B) Percentage of time spent exploring the open arms in the EPMT; (C) Freezing time during the re-exposure phase of the FCT; (D) Freezing time in the 24 h post-fear test; (E) The extinction coefficient; (F) Total motion trajectory diagram for the OFT (Tukey’s test).
